# Long noncoding RNA OR3A4 promotes metastasis and tumorigenicity in gastric cancer

**DOI:** 10.18632/oncotarget.7217

**Published:** 2016-02-06

**Authors:** Xiaobo Guo, Ziguo Yang, Qiaoming Zhi, Dan Wang, Lei Guo, Guimei Li, Ruizhen Miao, Yulong Shi, Yuting Kuang

**Affiliations:** ^1^ Department of Gastrointestinal Surgery, Shandong Provincial Hospital Affiliated to Shandong University, Jinan 250021, China; ^2^ Departments of General Surgery, The First Affiliated Hospital of Soochow University, Suzhou 215006, China; ^3^ Departments of Science and Education, Shandong Provincial Hospital Affiliated to Shandong University, Jinan 250021, China; ^4^ Department of Pediatrics, Shandong Provincial Hospital Affiliated to Shandong University, Jinan 250021, China; ^5^ Department of Hepatobiliary Surgery, Eastern Hepatobiliary Surgery Hospital, Second Military Medical University, Shanghai 200438, China

**Keywords:** gastric cancer, lncRNA, metastasis, OR3A4, tumorigenesis

## Abstract

The contribution of long noncoding RNAs (lncRNAs) to metastasis of gastric cancer remains largely unknown. We used microarray analysis to identify lncRNAs differentially expressed between normal gastric tissues and gastric cancer tissues and validated these differences in quantitative real-time (qRT)-PCR experiments. The expression levels of lncRNA olfactory receptor, family 3, subfamily A, member 4 (OR3A4) were significantly associated with lymphatic metastasis, the depth of cancer invasion, and distal metastasis in 130 paired gastric cancer tissues. The effects of OR3A4 were assessed by overexpressing and silencing OR3A4 in gastric cancer cells. OR3A4 promoted cancer cell growth, angiogenesis, metastasis, and tumorigenesis *in vitro* and *in vivo*. Global microarray analysis combined with RT-PCR, RNA immunoprecipitation, and RNA pull-down analyses after OR3A4 transfection demonstrated that OR3A4 influenced biologic functions in gastric cancer cells via regulating the activation of PDLIM2, MACC1, NTN4, and GNB2L1. Our results reveal OR3A4 as an oncogenic lncRNA that promotes tumor progression, Therefore, lncRNAs might function as key regulatory hubs in gastric cancer progression.

## INTRODUCTION

The incidence of gastric cancer has decreased dramatically over the last 50 years; however, according to the International Agency for Research on Cancer (IARC)-Globocan 2008 findings, gastric cancer remains the third most frequent cause of cancer-related deaths after lung and liver cancers in male individuals and after breast and lung cancers in female individuals [1–3]. Many patients with gastric cancer have progressed beyond the opportunity for curative treatment at the time of diagnosis, and have an extremely poor prognosis [3]. Several molecular alterations present in gastric cancer have been characterized [4–6]. However, many important questions, including the mechanisms underlying gastric cancer metastasis remain unanswered.

The human transcriptome contains not only large numbers of protein-coding messenger RNAs (mRNAs), but also a large set of non-protein-coding transcripts that have structural, regulatory, or unknown functions [7–9]. While studies of small noncoding RNAs (18–200 nucleotides in length) have dominated the field of RNA biology in recent years [10, 11], a surprisingly wide array of cellular functions is associated with long noncoding RNAs (lncRNAs). Altered expression of lncRNAs has been documented in multiple types of human cancer, prompting increased interest in their potential use as biomarkers for diagnosis and prognosis, in addition to their potential roles as therapeutic targets [12–16]. Acculating evidence demonstrates that lncRNAs play a pivotal role in promoting cancer metastasis, for example lncRNA MALA-1 in lung cancer [14, 17, 18]. However, whether lncRNAs play a role in metastasis of gastric cancer and remains largely unknown.

We used a custom lncRNA microarray platform containing probes for lncRNAs expressed from the human genome in addition to a selected set of cancer metastasis-related lncRNAs and protein-coding genes to generate expression profiles from a collection of gastric cancer and normal tissues. Expression of lncRNA subsets was detected across all samples tested. We identified expression signatures comprising lncRNAs that are significantly correlated with gastric cancer metastasis. Expression of OR3A4 (Accession Number: NR_024128.1) was detected in both primary gastric cancer tissues, and the peripheral blood of gastric cancer patients. We also found that OR3A4 promoted the growth, invasion, metastasis, and tumorigenesis of gastric cancer cells, both *in vitro* and *in vivo*. We identified several OR3A4 target genes by global microarray analysis following OR3A4 overexpression, combined with RT-PCR, RNA immunoprecipitation, and RNA pull-down analyses. Our data suggest that OR3A4 is modulated during tumorigenesis and tumor progression and may participate in molecular interactions relevant to malignant transformation and metastasis in gastric cancer.

## RESULTS

### Changes in lncRNA OR3A4 expression in gastric cancer identified by expression profiling

**Figure 1:**
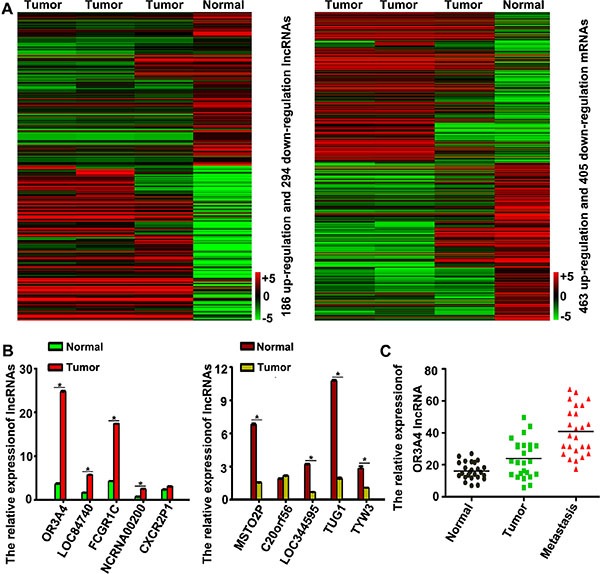
Hierarchical clustering of lncRNA expression in gastric cancer tissues and normal gastric tissue (**A**) Hierarchical clustering showed systematic variations in the expression of lncRNAs and protein-coding RNAs in 3 tissues, compared with those observed in gastric cancer tissues and normal tissues. The threshold set for upregulated and downregulated lncRNAs was a fold change ≥ 6 and a *P* value ≤ 0.05. A total of 186 lncRNAs were upregulated and 294 were downregulated in gastric cancer samples, compared with normal gastric tissue samples. The key color bar indicates that lncRNAs and mRNA expression levels increased from a green to a red color, compared with those of the controls (a dark color indicates that the expression level is close to that of control). (**B**) Validation of the differential expression of 10 metastasis-related lncRNAs in 3 gastric cancer tissues and 3 normal gastric tissues by qRT-PCR. (**C**) OR3A4 expression in 25 normal gastric tissues, 25 gastric cancer tissues, and 25 corresponding metastatic gastric cancer tissues. Each bar represents the mean ± the standard deviation from 3 independent experiments. *P < 0.05.

Hierarchical clustering revealed systematic variations in the expression of lncRNAs and protein-coding RNAs in 3 gastric cancer tissues, compared with normal tissues (Supplementary Table S1, Figure 1A). The microarray data set was deposited in the GEO database (http://www.ncbi.nlm.nih.gov/gds/?term=GSE54835). Based on expression differences (≥ 6-fold) observed between gastric cancer tissues and normal gastric tissue, our data revealed 186 upregulated and 294 downregulated lncRNAs that were expressed abnormally in gastric carcinoma samples (Figure 1A). We next validated the microarray-analysis findings, findings randomly selecting 10 metastasis-related lncRNAs from the differentially expressed lncRNAs and analyzing their expression in gastric cancer and normal tissues by qRT-PCR (Table 1, Figure 1B). OR3A4, LOC84740, FCGR1C, and NCRNA00200 were upregulated in gastric cancer tissues, whereas MSTO2P, LOC344595, TUG1, and TYW3 were downregulated (*P* < 0.05).

**Table 1:** Elective lncRNAs in lncRNAs microarray

lncRNA	Down or Up	Fold Change	Official Full Name	Location	Genbank Number	Relationship
OR3A4	up	55.87354	olfactory receptor, family 3, subfamily A, member 4 pseudogene	Chr17p13.3	NR_024128	intergenic
LOC84740	up	45.560715	AFAP1 antisense RNA 1	Chr4p16.1	NR_026892	natural antisense
FCGR1C	up	18.387403	Fc fragment of IgG, high affinity Ic, receptor (CD64), pseudogene	Chr1q21.2	NR_027484	intergenic
NCRNA00200	up	37.474087	long intergenic non-protein coding RNA 200	Chr10p15.3	NR_015376	intergenic
CXCR2P1	up	27.404158	chemokine (C-X-C motif) receptor 2 pseudogene 1	Chr2q35	NR_002712	intergenic
MSTO2P	down	−1368.9837	misato homolog 2 pseudogene	Chr1q22	NR_024117	natural antisense
C20orf56	down	−33.629803	long intergenic non-protein coding RNA 261	Chr20p11.21	NR_001558	intergenic
LOC344595	down	−58.107555	long intergenic non-protein coding RNA 883	Chr3q13.12	NR_028301	intergenic
TUG1	down	−24.081228	taurine upregulated 1	Chr22q12.2	NR_002323	intergenic
TYW3	down	−85.79346	tRNA-yW synthesizing protein 3 homolog	Chr1p31.1	NM_001162916	exon sense-overlapping

**Figure 2:**
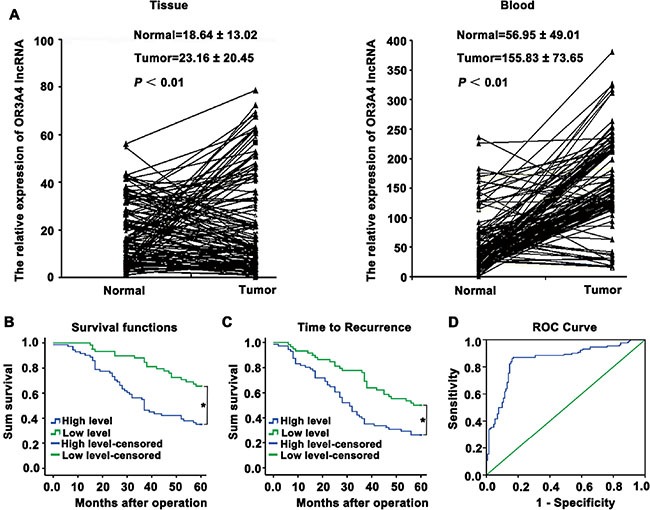
Upregulation of OR3A4 expression in primary tumor tissues and peripheral blood from gastric carcinoma patients, compared with the corresponding controls (**A**) qRT-PCR analysis of OR3A4 expression in primary tumor tissues and peripheral blood samples from 130 gastric carcinoma patients and healthy controls. The mean and standard deviation of OR3A4 expression levels are shown. (**B**) Kaplan–Meier analysis of overall survival curves for patients with gastric cancer, according to the level of OR3A4 expression. (**C**) Time to recurrence of the high-OR3A4 expression group was significantly shorter than that of the low-OR3A4 expression group. (**D**) Receiver operating characteristics curve of blood DNA detection for OR3A4. The AUC was 0.852 ± 0.025 (*P* < 0.01), with a sensitivity and specificity of 86.94% and 91.27%, respectively. *P < 0.05.

We next investigated the role of OR3A4 in gastric cancer, by examining a panel of 25 matched sets of normal gastric tissue, gastric cancer tissue, and corresponding metastatic tissue. OR3A4 expression was upregulated in metastatic tissues compared with the corresponding normal gastric tissues and gastric cancer tissues (Figure 1C, *P* < 0.05). It is noteworthy that the expression levels of OR3A4 were significantly associated with gastric cancer metastasis and that OR3A4 expression in gastric cancer tissues was related to clinicopathological characteristics, and OR3A4 expression levels were significantly associated with metastasis. We then investigated relationships between OR3A4 expression and the clinicopathological factors using 130 paired gastric cancer tissues from patients with gastric cancer. Expression level was significantly associated with lymphatic metastasis, the depth of cancer invasion, and distal metastasis (Table 2, *P* < 0.05). However, OR3A4 expression levels were not associated with patient sex or age, tumor sizes, cell differentiation, gross appearances, the tumor sites, or the tumor-node-metastasis (TNM) stage. We further examined OR3A4 expression levels in primary tumor tissues and associated plasma DNA from 130 patients with gastric carcinoma and healthy controls by qRT-PCR. The mean expression levels of OR3A4 in 130 primary tumor tissues and in plasma DNA were 23.16 ± 20.45% and 155.83 ± 73.65%, respectively (Figure 2A, *P* < 0.01). Significant overexpression of OR3A4 was observed in the cancer group compared with healthy controls. Overall survival curves and time-to-recurrence (TTR) curves were plotted according to the OR3A4 expression level using the Kaplan–Meier method. The high-OR3A4 expression group had significantly shorter overall survival and TTR than did the low-OR3A4 expression group (*P* < 0.05; Figure 2B and 2C). Furthermore, the receiver operating characteristics curve yielded an area under the curve (AUC) for detecting OR3A4 expression in blood DNA of 0.852 ± 0.025, which was significantly higher than that of the null hypothesis (true area: 0.5; Figure 2D, *P* < 0.01). These data suggested that OR3A4 expression in the blood could serve as a molecular marker for gastric cancer. Youden's index was then used to identify an optimal cut-off point for the detection of gastric cancer, with an optimal operating point for the blood expression level of OR3A4 of 86.94%, and a specificity of 91.27%. Taken together, these data suggest an important role for OR3A4 in gastric cancer.

**Table 2:** Clinicopathological features and OR3A4-lncRNA expression in gastric cancer patients

Factors	No. of patients	Mean expression ofOR3A4-lncRNA (mean ±SD)	*P*-value
Age (year)			
< 65	87	26.14 ± 1.65	1.573
≥ 65	43	18.24 ± 2.59	
Gender			
Male	76	21.27 ± 6.58	0.782
Female	54	14.26 ± 4.47	
Cell differentiation			
Poor differentiation	83	21.51 ± 6.17	1.047
Moderate differentiation	47	17.26 ± 5.41	
Tumor size			
< 5 cm	78	14.57 ± 2.16	3.632
≥ 5 cm	52	16.58 ± 7.82	
Gross appearance			
Borrmann I + II type	47	21.06 ± 2.71	0.683
Borrmann III + IV type	83	27.17 ± 3.18	
Site of tumor			
Cardia	35	16.50 ± 2.68	1.87
Body	52	23.26 ± 3.79	
Antrum	43	18.21 ± 7.63	
Lymphatic metastasis			
Positive	86	23.16 ± 5.21	0.036*
Negative	44	16.53 ± 7.31	
Depth of cancer invasion			
T2	24	9.52 ± 6.09	0.028*
T3	67	27.02 ± 5.21	
T4	39	15.06 ± 2.41	
TNM^#^ Stage			
I	13	8.14 ± 2.16	0.385
II	29	14.21 ± 1.56	
III	67	16.05 ± 1.41	
IV	21	29.17 ± 6.73	
Distal metastasis			
Positive	48	25.41 ± 5.09	0.032*
Negative	82	18.16 ± 2.62	

# TNM, tumor-node-metastasis; *p < 0.05.

### Enhanced OR3A4 expression in gastric cancer cells promoted cell migration and invasion *in vitro*

**Figure 3:**
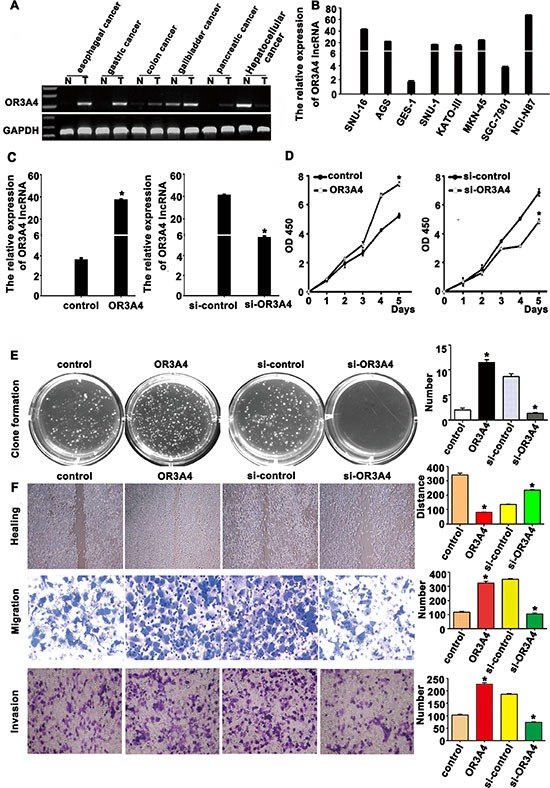
OR3A4 promotes proliferation, migration, and invasion in gastric cancer cells (**A**) Semi-quantitative qRT-PCR analysis of OR3A4 expression in primary tumor tissues and healthy control tissues. (**B**) OR3A4 expression levels in 7 gastric cancer cell lines and GES-1 cells by qRT-PCR. (**C**) qRT-PCR analysis confirmed OR3A4 overexpression in SGC-7901 cells stably transfected with a pEGFP-N1-OR3A4 expression vector versus cells transfected with the vector control. QRT-PCR was also used to confirm specific OR3A4 silencing in NCI-N87 cells transfected with a pSilencer-OR3A4 siRNA vector, compared with the si-control. (**D, E**) OR3A4 overexpression promotes cell proliferation and Colony formation in soft agar in SGC7901 cells, compared with control group. (**F**) Migratory and invasive capacities in gastric cancer cells increased after OR3A4 overexpression, as determined by performing scratch-healing, transwell-migration, and transwell-invasion assays.

We next used semi-quantitative RT-PCR to assess OR3A4 expression in tissues from patients with esophageal, gastric, colon, gallbladder, pancreatic, and hepatocellular cancers. With the exception of hepatocellular cancer, OR3A4 expression levels were higher in the cancer tissues compared with matched normal tissues (Figure 3A). QRT-PCR analysis revealed that OR3A4 was highly expressed in 7 different gastric cancer cell lines (SNU-16, AGS, SNU-1, KATOIII, MKN45, NCI-N87, and SGC7901) and 1 immortalized gastric mucosa cell line (GES-1) (Figure 3B). In particular, OR3A4 expression increased 39.88-fold, 25.22-fold, and 12.87-fold in the NCI-N87, SUN-16, and AGS cell lines, respectively. These results suggested that OR3A4 may play a role in gastric cancer carcinogenesis. To evaluate the effects of OR3A4 on the biological behavior of gastric cancer cell lines, we constructed a stable pcDNA-OR3A4 expression vector and a stable pSilencer-OR3A4 small-interfering RNA (siRNA) vector and transfected gastric cancer cell lines SGC-7901 and NCI-N87 with these constructs. QRT-PCR confirmed that OR3A4 expression levels were upregulated or downregulated (Figure 3C, *P* < 0.01), compared with those observed in control transfectants. Cell-Counting Kit-8 assays revealed increased cell proliferation in SGC7901 cells transfected with the OR3A4-expressing vector, compared with control cells (Figure 3D, left, *P* < 0.01). Meanwhile, siRNA-mediated OR3A4 knockdown significantly suppressed NCI-N87 cells growth, compared with that observed in controls group (Figure 3D, right, *P* < 0.01). Consistent with the enhanced cell proliferation, SGC-7901 cells overexpressing OR3A4 had significantly higher levels of proliferating cell nuclear antigen (PCNA) expression than did control cells (Supplementary Figure S1A). Contrastingly, OR3A4 downregulation in NCI-N87 cells inhibited proliferation and PCNA expression (Supplementary Figure S1A). Similar findings were obtained using a colony-formation assays, in which gastric cancer cells transfected with the OR3A4-expression vector or the OR3A4-siRNA vector were grown on soft agar. The colony-formation rate of SGC-7901 cells was 11.50% ± 0.57% in the OR3A4 group and 2.00% ± 0.40% in the control group (Figure 3E, *P* < 0.001). Taken together, these results suggest that OR3A4 has a physiological role in regulating cell proliferation.

We also evaluated the cell migration and invasion capacities of gastric cancer cells with modified expression of OR3A4 using a scratch-healing assays (Figure 3F, upper panels), a transwell-migration assays (Figure 3F, middle panels), and a transwell-invasion assays (Figure 3F, lower panels). Overexpressing OR3A4-transfected SGC-7901 cells almost completely closed the wound at 48 h post-scratching, whereas cells transfected with the empty vector were unable to heal the wound. The mean wound distances of the experimental samples and control samples at 48 h were significantly different (28.35 ± 4.75 μm vs. 251.01 ± 2.42 μm, *P* < 0.001; Figure 3F). The migration ability of SGC-7901 cells overexpressing OR3A4 was significantly enhanced, compared with that of control transfectants (323.52 ± 10.56 vs. 117.67 ± 5.26, *P* < 0.001; Figure 3F). The number of invasive cells in the OR3A4-transfected sample also significantly increased compared with the control transfectants (327.35 ± 5.45 vs. 140.40 ± 2.79, *P* < 0.001; Figure 3F). We also assessed the performance of NCI-N87 cells following OR3A4 knockdown using scratch-healing, transwell-migration, and transwell-invasion assays. Results from these assays followed an opposite trend to those described above for the SGC-7901 cells that overexpressed OR3A4, suggesting that OR3A4 promotes the migration and invasion in gastric cancer cells.

### Enhanced OR3A4 expression in gastric cancer cells promoted tubule formation in human umbilical vein endothelial cells (HUVECs)

**Figure 4:**
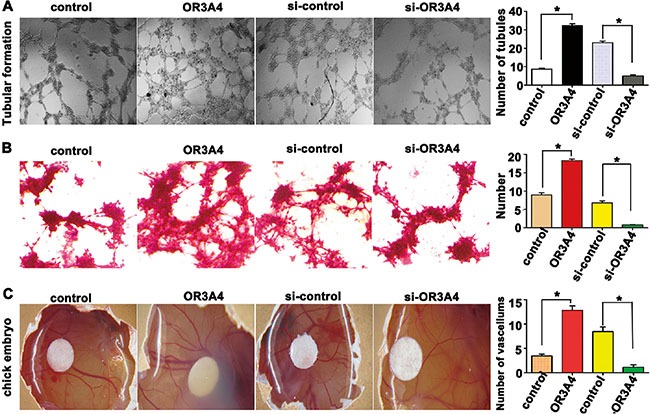
OR3A4 induces tubular formation and promoted angiogenesis in chicken embryos (**A**) The number of tubules formed by human umbilical vein endothelial cells treated with supernatants from SCG-7901/OR3A4 cells or NCI-N87-knockdown cells was assessed using light microscopy. (**B**) Vasculogenic mimicry was assessed as PAS-positive extracellular matrix surrounded by cancer cells. (**C**) A traditional chick embryo chorioallantoic membrane assay was used to observe the effect of restoring OR3A4 on angiogenesis. The eggs were photographed, and the number of blood vessels surrounding the filter paper disc was assessed. Each bar represents the mean value ± standard deviation from 3 independent experiments. *P < 0.05.

HUVECs (2 × 10^4^ cells/well) were resuspended in supernatants from SCG-7901/OR3A4 or SGC-7901/control cells. Supernatant from SCG-7901/OR3A4 cells showed a strong positive effect on tubule formation of HUVECs, compared with control supernatant (32.32 ± 0.92 vs. 8.69 ± 0.47, *P* < 0.001; Figure 4A). In contrast, supernatant from OR3A4-knockdown NCI-N87 cells impaired tubular formation by HUVECs (4.99 ± 0.62 vs. 23.03 ± 0.89, *P* < 0.001; Figure 4A).

### Enhanced OR3A4 expression in gastric cancer cells promoted vasculogenic mimicry

SCG-7901/OR3A4 and SGC-7901/control cells (1.5 × 10^5^ cells) were plated on 3-dimensional (3D) gels. After culturing for 4 days without changing the culture media, vasculogenic mimicry was assessed by measuring the presence of a periodic acid-Schiff (PAS)-positive extracellular matrix (ECM) surrounded by cancer cells. SCG-7901/OR3A4 cells strongly promoted vasculogenic mimicry formation with respect to the tubular length and formation of tubular intersecting nodes, compared with the control cells (18.32 ± 0.40 vs. 8.95 ± 0.60, *P* < 0.001; Figure 4B). Consistently, NCI-N87 cells with depleted OR3A4 exhibited strong suppression of vasculogenic mimicry in terms of both the tubular length and formation of tubular intersecting nodes, compared with controls (0.80 ± 0.07 vs. 6.79 ± 0.55, *P* < 0.001; Figure 4B).

### Enhanced OR3A4 expression in gastric cancer cells promoted angiogenesis in chicken embryos

A traditional chicken embryo chorioallantoic membrane (CAM) assay was employed to evaluate the effects of OR3A4 expression on angiogenesis. Each day, a 30-mL supernatant sample from SCG-7901/OR3A4 and SGC-7901/control cells was dropped onto the surface of the CAM, which covered a piece of a sterilized filter paper disc. After 3 days, the eggs were photographed and blood vessels surrounding the filter paper disc were counted. The number of blood vessels significantly increased in eggs treated with the SCG-7901/OR3A4 supernatant compared with control eggs (12.85 ± 0.89 vs. 3.47 ± 0.35, *P* < 0.001; Figure 4C). Supernatant from NCI-N87 gastric cancer cells with depleted OR3A4 consistently displayed a strongly inhibitory effect on angiogenesis in chicken embryos, compared with the control embryos (number of tubules 1.12 ± 0.52 vs. 8.45 ± 0.98, *P* < 0.001; Figure 4C). These data reveal that upregulation of OR3A4 expression in gastric cancer cell lines promotes angiogenesis in chick embryos. Based on the above findings, we suspected that upregulation of OR3A4 expression could induce expression of the vascular endothelial growth factor C (*VEGF-C*) and matrix metallopeptidase 9 (*MMP9*) genes in gastric cell lines. Indeed, our qRT-PCR data showed that *VEGF-C* and *MMP9* expression increased following OR3A4 overexpression (Supplementary Figure S1B) and decreased following OR3A4 knockdown (Supplementary Figure S1B). These results suggested that OR3A4 contributes to angiogenesis.

### Effect of overexpressing OR3A4 on tumorigenicity and peritoneal spreading in nude mice

**Figure 5:**
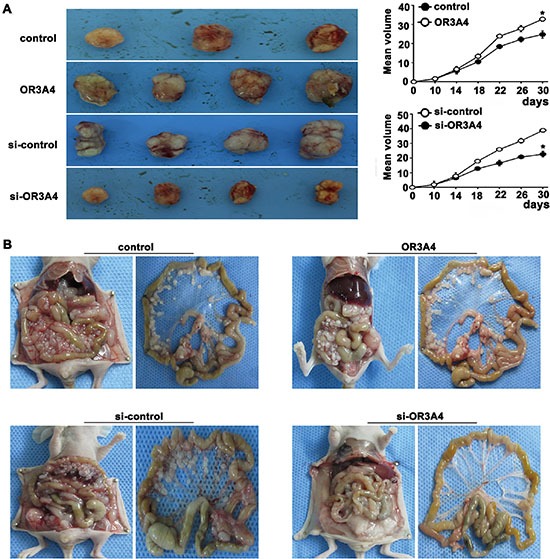
OR3A4 promotes tumorigenicity and metastasis of gastric cancer cells in nude mice (**A**) Photographs showing tumor progression in nude mice. Tumor diameters were measured every 3 days. (**B**) Effect of OR3A4 overexpression or knockdown in the peritoneal cavity of nude mice on peritoneal spreading. Each bar represents the mean value ± standard deviation from 3 independent experiments. *P < 0.05.

We next examined the effect of OR3A4 overexpression on tumorigenicity *in vivo* by subcutaneously inoculating SCG-7901/OR3A4 and SGC-7901/control cells into the flank regions of nude mice. OR3A4-overexpressing cells showed significantly greater tumorigenicity. Rapid tumor growth was observed in the SCG-7901/OR3A4 group after 1 month compared with the SGC-7901/control group (32.73 ± 0.13 vs. 24.73 ± 0.95, *P* < 0.005; Figure 5A). OR3A4 overexpression in the peritoneal cavity of nude mice (by intraperitoneal injection) promoted peritoneal spreading (Figure 5B). OR3A4 knockdown in NCI-N87 cells had the opposite effect, suppressing tumorigenicity and peritoneal spreading (22.61 ± 1.95 vs. 38.91 ± 1.13, *P* < 0.005; Figure 5A). These data demonstrate the *in vivo* tumor-promoting role of OR3A4 expression in gastric cancer cells. QRT-PCR analysis also showed that the *VEGF-C*, *Ki-67*, and *MMP9* genes were highly expressed in tumors derived from OR3A4-overexpressing compared with control transfectants (Supplementary Figure S1C). These findings indicate that OR3A4 regulated tumorigenicity and peritoneal spreading of gastric cancer, both *in vitro* and *in vivo*.

### Identification of OR3A4 target genes

**Figure 6:**
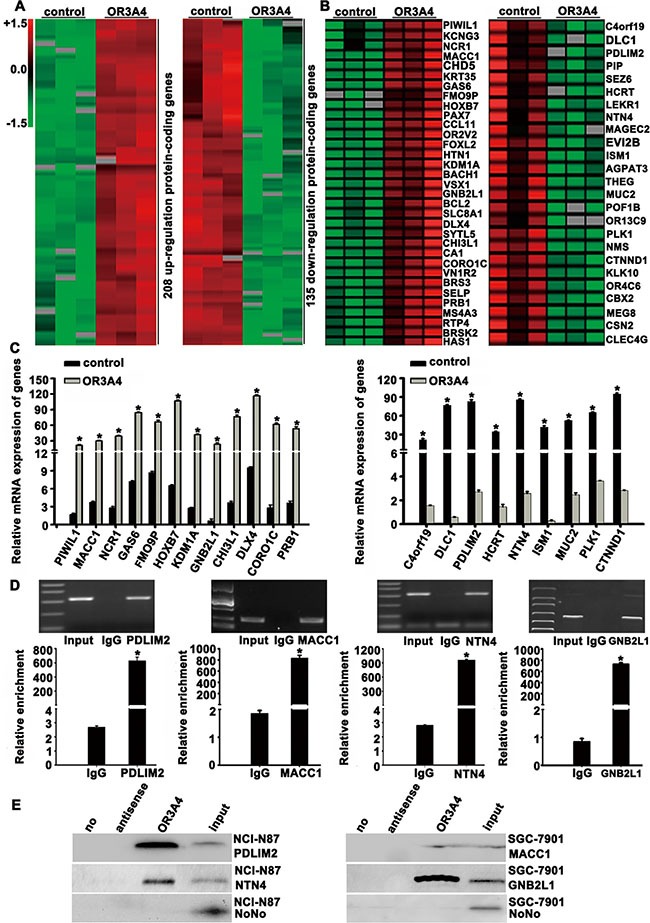
Target gene identification by global microarray analysis (**A**) Clustering map of differentially expressed genes that overlapped with the cancer-associated gene set in the Molecular Signatures Database. Our results identified 208 genes that were upregulated and 135 that were downregulated, using a fold-change cutoff of > 3. (**B**) We selected 58 cancer-associated genes including 33 upregulated genes and 25 downregulated genes for cluster mapping with the MeV microarray-analysis platform. Rows represent genes and columns represent experimental cells. Upregulated genes are shown in red and downregulated genes are shown in green. (**C**) Confirmation of the upregulation of *PIWIL1*, *MACC1*, *NCR1*, *GAS6*, *FMO9P*, *HOXB7*, *KDM1A*, *GNB2L1*, *CHI3L1*, *DLX4*, *CORO1C*, and *PRB1* expression (left) and downregulation of *C4orf19*, *DLC-1*, *PDLIM2*, *HCRT*, *NTN4*, *ISM1*, *MUC2*, *PLK1*, and *CTNND1* expression (right). (**D**) RNA immunoprecipitation was performed using antibodies against PDLIM2, MACC1, NTN4, and GNB2L1. OR3A4 RNA levels were quantified using a specific primer. (**E**) RNA pull-down was performed as described in the Materials and Methods section. OR3A4 RNA was incubated with nuclear extracts and the levels of bound PDLIM2, MACC1, NTN4, or GNB2L1 protein were assayed by western blotting. Each bar represents the mean value ± standard deviation from 3 independent experiments. *P < 0.05.

We next investigated the molecular mechanisms underlying the promotion of cell growth, migration, invasion, angiogenesis, tumorigenicity, and metastasis of gastric cancer by OR3A4, we analyzed the genome-wide transcriptome profiles of SCG-7901/OR3A4 and SGC-7901/control cells using an Agilent oligo microarray. Based on expression differences (fold change > 3) between SCG-7901/OR3A4 and SGC-7901/control cells, we identified 208 genes that were upregulated and 135 genes that were downregulated upon OR3A4 overexpression (Figure 6A). We then searched for genes that overlapped with cancer-associated and molecular function-related gene sets deposited in MSigDB (C4 and C5 gene sets; http://www.broad.mit.edu/gsea/msigdb/index.jsp) and selected 58 cancer-associated genes (33 upregulated genes and 25 downregulated genes) for cluster mapping on the MeV microarray analysis platform (www.tm4.org/mev.html, Figure 6B). QRT-PCR analysis performed to verify these genes (Table 3) confirmed our microarray findings for 12 upregulated genes and 9 downregulated genes (Figure 6C). The gene names and functional annotations are listed in Table 3. To determine whether these genes are direct target genes of OR3A4, we performed RNA immunoprecipitation assays using an antibody against the test gene product and nuclear extracts from SGC-7901 or NCI-N87 cells. OR3A4 was significantly enriched in extracts obtained using antibodies against PDLIM2, MACC1, NTN4, and GNB2L1 (Figure 6D). These findings are supported by RNA pull-down experiments that demonstrated significant enrichment of *PDLIM2*, *MACC1*, *NTN4*, and *GNB2L1* with OR3A4 RNA compared with negative control antisense RNA (Figure 6E). Therefore, these genes are direct targets of OR3A4. Together, these data indicated that OR3A4 upregulation contributes to metastasis and tumorigenesis in gastric cancer by regulating the activation of PDLIM2, MACC1, NTN4, and GNB2L1.

**Table 3:** Verified genes and their functions by real-time PCR

Genes	Accession number	Functions
Upregulated		
PIWIL1	NM_001190971	an key molecular factor along with the tumor occurrence and development, a potential biomarker for prognosis evaluation of gastric cancer
MACC1	NM_182762	Cellular growth, epithelial-mesenchymal transition, angiogenesis, cell motility, invasiveness, and metastasis.
NCR1	NM_001145457	NCR1 expression and poor prognosis factors such as low haemoglobin level, high lymphocyte count or elevated C-reactive protein
GAS6	NM_000820	the stimulation of cell proliferation and may play a role in thrombosis
FMO9P	NR_002925	flavin containing monooxygenase 9 pseudogene
HOXB7	NM_004502	cell proliferation and differentiation; melanoma and ovarian carcinoma
KDM1A	NM_001009999	encodes a nuclear protein containing a SWIRM domain, a FAD-binding motif, and an amine oxidase domain.
GNB2L1	NM_006098	function as an internal factor involved in the growth and survival of hepatocellular carcinoma;positively regulated cell migration and proliferation
CHI3L1	NM_001276	the process of inflammation and tissue remodeling.
DLX4	NM_001934	DLX4 induces cancer cells to undergo epithelial to mesenchymal transition through TWIST, enhancing tumor migration, invasion and metastasis
CORO1C	NM_001105237	cell cycle progression, signal transduction, apoptosis, and gene regulation.
PRB1	NM_005039	This gene encodes a precursor for proline-rich salivary proteins.
Downregulated		
C4orf19	NM_001104629	Genetic variation influences glutamate concentrations in brains of patients with multiple sclerosis
DLC-1	NM_001164271	functions as a tumor suppressor gene in a number of common cancers, including prostate, lung, colorectal, and breast cancers
PDLIM2	NM_021630	Cell migration and adhesion through interactions with the actin cytoskeleton via the PDZ domain. a putative tumor suppressor protein
HCRT	NM_001524	feeding behavior, metabolism, and homeostasis.
NTN4	NM_021229	proliferation, migration, adhesion, tube formation and survival of human lymphatic endothelial cells.
ISM1	NM_080826	angiogenesis inhibitor
MUC2	NM_002457	recurrence in colorectal carcinoma; increased from well differentiated to moderately differentiate to poorly differentiate in gastric adenocarcinoma.
PLK1	NM_005030	Breast cancer patients expressing elevated PLK1 expression show significantly reduced survival.
CTNND1	NM_001085458	The function of this gene in adhesion between cells and signal transduction.

### Functional validation of the novel OR3A4 downstream target PDLIM2

**Figure 7:**
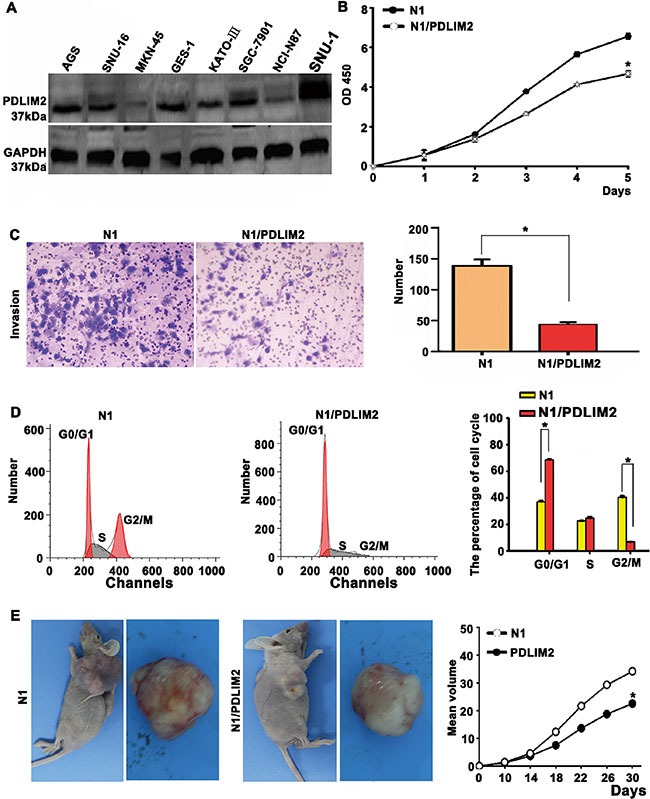
Functional validation of PDLIM2 (a novel downstream target of OR3A4) in MKN-45 cells (**A**) Expression levels of the *PDLIM2* gene in gastric cancer cells. (**B**) Overexpression of PDLIM2 inhibited cell growth in MKN-45 cells, as assessed by performing CCK-8 assays. (**C**) Ectopic PDLIM2 expression inhibited the invasion of MKN-45 cells, as determined in an invasion assay. (**D**) PDLIM2-overexpressing MKN-45 cells showed an increased percentage of cells in the G0/G1 phase and decreased percentages of cells in the S and G2/M phases. (**E**) Restoration of PDLIM2 expression inhibited tumorigenesis *in vivo*. Each bar represents the mean value ± standard deviation from 3 independent experiments. *P < 0.05.

We selected PDLIM2, a novel downstream target of OR3A4, for further functional validation. First, we screened several gastric cancer cell lines for PDLIM2 expression by western blotting. Different levels of PDLIM2 expression were detected among 8 cell lines (Figure 7A) and we selected MKN-45 and NCI-N87 cells for subsequent transfection studies to analyze the effect of PDLIM2 on cell proliferation and invasion. Ectopic PDLIM2 expression in MKN-45 cells significantly inhibited cell growth and invasion as early as 120 h post-transfection compared with that observed in empty vector-transfected MKN-45 cells (*P* < 0.05; Figure 7B and 7C). Ectopic PDLIM2 expression caused a significant decrease in the percentage of G2/M phase cells and a higher percentage of G0/G1 phase or S phase cells, compared with MKN-45/N1-transfected cells, as determined by flow cytometry (*P* < 0.05; Figure 7D), These findings suggest that PDLIM2 overexpression promoted the G2/M transition. Additionally, tumorigenicity was significantly reduced in PDLIM2-transfected cells 1 month after transfection, whereas rapid tumor growth was observed in the control groups (*P* < 0.05; Figure 7E). We also assessed cell growth (Supplementary Figure S2A), invasion (Supplementary Figure S2B), cell cycle progression (Supplementary Figure S2C), and tumorigenicity (Supplementary Figure S2D) following PDLIM2 overexpression in NCI-N87 gastric cancer cells and obtained similar results to those described for MKN-45 cells. These results suggested that the molecular mechanism by which OR3A4 promotes tumorigenicity and peritoneal spreading of gastric cancer involves PDLIM2 downregulation.

## DISCUSSION

Mounting evidence suggests that the molecular mechanisms underlying carcinogenesis involve both protein-coding genes, and noncoding regulatory RNAs [19–21]. Some lncRNAs play a pivotal role in the occurrence of gastric cancers [17, 22, 23]. Additionally, numerous lncRNAs are deregulated in various solid tumors, and several lncRNAs can regulate cancer metastasis by directly targeting chromatin-modification complexes [9, 18]. However, few lncRNAs have been functionally studied in detail and many important questions remain to be addressed.

We used a custom lncRNA microarray platform with probes for metastasis-related lncRNAs expressed from the human genome. We also studied a selected set of cancer-related protein-coding genes to identify non-overlapping expression signatures of a small number of lncRNAs that are aberrantly expressed in human gastric cancer, compared with normal, matched tissues. While the results of our lncRNA microarray are slightly different from those of others who have performed similar studies in gastric cancer (GSE54835), the overall trend is the same. Based on the microarray results we randomly selected 5 lncRNAs that were upregulated and 5 that were downregulated and investigated their expression levels in human gastric cancer tissues and normal tissues. OR3A4, LOC84740, FCGR1C, and NCRNA00200 were overexpressed in gastric cancer tissues, whereas MSTO2P, LOC344595, TUG1, and TYW3 were downregulated. Of these, OR3A4 showed the highest expression level and was upregulated in gastric cancer and metastatic lymph node tissues. Therefore, OR3A4 may have an important role in the malignant transformation and the metastasis of gastric cancer.

The expression of OR3A4 in gastric carcinoma and its correlation with patient clinicopathological features was poorly characterized prior to our current study. Therefore, we examined OR3A4 expression levels and the clinicopathological characteristics of 130 patients with gastric cancer. Although no relationship was observed between OR3A4 expression and patient sex and age, tumor size, cell differentiation, gross appearance, site of tumor, or TNM stage, there were significant associations between OR3A4 expression and lymphatic metastasis, the depth of cancer invasion, and distal metastasis. These results indicate that the OR3A4 expression level might influence tumor progression during gastric cancer development. In clinical practice, patients with gastric cancer are often not symptomatic in the early stages of the disease. Therefore, serum screening for OR3A4 represents a promising diagnostic tool because it is easy and inexpensive. Significant OR3A4 overexpression was detected in the peripheral blood of gastric cancer patients, compared with that observed in healthy control subjects. According to Youden's index, the optimal operating point of the blood expression level of OR3A4 was 86.94%, and the specificity was 91.27%. We found that patients with high OR3A4 expression had an increased risk of recurrence and a significantly reduced overall post-operative survival. Thus, the OR3A4 expression level in gastric cancer tissues and peripheral blood may serve as a valuable prognostic indicator for patients with gastric cancer.

Our data clearly showed that OR3A4 is highly expressed in gastric cancer tissues from patient samples and in gastric cancer cell lines. The effects of OR3A4 were assessed *in vitro* and *in vivo* by overexpressing and silencing the lncRNA. Cell-Counting Kit-8 assay results indicated that cell proliferation increased in SGC-7901 cells upon forced OR3A4 overexpression and decreased in NCI-N87 cells upon OR3A4 silencing. We also measured the expression levels of PCNA, which increases the processivity of leading strand synthesis during DNA replication [24, 25]. PCNA was expressed at significantly higher levels upon OR3A4 overexpression in gastric cancer cells, compared with control cells. Our findings suggest that OR3A4 plays a physiological role in regulating cell proliferation. Tumor spreading and metastasis are influenced by tumor migration and invasion capacity, both of which were promoted by OR3A4 *in vitro*. A rich blood supply is requires for tumor growth, spread, and metastasis. The tube-formation assay used in this study is based on the ability of endothelial cells to form 3D capillary-like structures when cultured in a gel containing basement membrane extract [26, 27]. Vasculogenic mimicry was first described by Maniotis et al. who characterized fluid-conducting channels by highly aggressive melanoma cells [28]. We found that OR3A4 overexpression effectively promoted the formation of capillary-and vasculogenic-mimicry structures. Additionally, results of a chicken CAM assay, an *in vivo* model for studying angiogenic activity [29], further, supported a pro-angiogenic role for OR3A4. Furthermore, enforced OR3A4 expression significantly upregulated *VEGF-C* and *MMP9* expression.

Currently, human tumor xenografts are the most widely used models for predicting antitumor efficacy. We demonstrated that increased tumorigenicity and peritoneal spreading of gastric cancer cells with increased OR3A4 expression in a nude mouse model. Additionally, enforced OR3A4 expression significantly promoted *VEGF-C*, *MMP9*, and *Ki-67* gene expression in the tumor tissues of nude mice. VEGF-C promotes angiogenesis and endothelial cell growth and can also affect the permeability of blood vessels [30, 31]; MMP9 regulates breakdown of the ECM during normal physiological processes [32, 33]; and Ki-67 is associated with, and may be necessary for, cellular proliferation [34]. These findings provide evidence that OR3A4 functions as a key mediator of cell growth, peritoneal spreading, and tumorigenicity and thus represents a promising target for gastric cancer treatment.

Microarray analysis is a powerful technology that allowed us to perform genome-wide analysis of expression profiles in a single experiment [4]. Here, we screened for target genes that are regulated by its overexpression. Specifically, we compared changes in the gene-expression profiles of gastric cancer cells (with or without OR3A4 overexpression) and identified a set of putative target genes. We further confirmed that 12 genes were upregulated and 9 were downregulated by qRT-PCR analysis, and subsequently applied RNA immunoprecipitation and RNA pull-down analysis to identify 4 direct targets—*PDLIM2*, *MACC1*, *NTN4*, and *GNB2L1*. PDLIM2 promotes cell migration and adhesion, and is also a putative tumor-suppressor protein [35, 36]; MACC1 regulates cellular growth, angiogenesis, motility, invasiveness, and metastasis [37–39]; NTN4 induces proliferation, migration, adhesion, tube formation, and survival [40]; and GNB2L1 regulates the growth and survival of hepatocellular carcinoma cells [41]. Therefore, OR3A4 overexpression may promote growth, invasion, metastasis, and tumorigenesis in gastric cancer *in vitro* and *in vivo* by upregulating MACC1 and GNB2L1 expression, and downregulating PDLIM2 and NTN4 expression.

We selected PDLIM2, a novel downstream target of OR3A4, for further functional validation and showed that ectopic PDLIM expression in gastric cells significantly inhibited cell growth and invasion. Ectopic PDLIM2 expression induced a lower percentage of G2/M-phase cells and a higher percentage of G0/G1-phase and S-phase cells, confirming that PDLIM2 overexpression promoted the G2/M transition. Additionally, tumorigenicity was significantly reduced in cells overexpressing PDLIM2. These results indicate that OR3A4 promote tumorigenicity and metastasis of gastric cancer, at least in part, by downregulating PDLIM2.

We used microarray analysis to identify differentially expressed between normal gastric tissues and gastric cancer OR3A4 was strongly expressed in gastric cancer tissues and matched peripheral blood samples. We used a vector-based overexpression or siRNA methods to demonstrate that OR3A4 promoted cell proliferation, migration, invasion, peritoneal spreading, and tumorigenesis both *in vitro* and *in vivo*. OR3A4 overexpression promotes tumorigenesis by modifying expression of a set of target genes that was identified by global microarray analysis and confirmed by qRT-PCR, RNA immunoprecipitation, and RNA pull-down assays. OR3A4 promotes the proliferation, migration, invasion, and tumorigenesis of gastric cancer cells by activating genes involved in cell migration and invasion (*MACC1*, *GNB2L1*, *DLX4*, *PDLIM2*, and *NTN4*), metastasis (*DLX4*), growth (*PIWIL1*, *MACC1*, *GAS6*, *HOXB7*, *GNB2L1*, and *DLC-1*), cellular adhesion (*PDLIM2*, *NTN4*, and *CTNND1*) and cell cycle progression (*CORO1C*). OR3A4 thus possesses important functions for the promotion of gastric cancer. Elevated OR3A4 expression in the peripheral blood of gastric cancer patients suggests its potential use as a biomarker for gastric cancer. Moreover, siRNA OR3A4 activity may serve as a potential strategy for gastric cancer treatment. More importantly, our data indicated that understanding the precise molecular mechanisms by which lncRNAs function in gastric cancer is critical for exploring potential new strategies for early diagnosis and therapy of gastric cancer.

## MATERIALS AND METHODS

### Cell lines and tissue samples

Gastric cancer cell lines MKN-45 and SGC-7901, an immortalized human gastric mucosa cell line GES-1, and HUVECs were provided by the Institute of Digestive Surgery of Ruijin Hospital (affiliated with Shanghai Jiao Tong University) [4, 5, 42–44]. NCI-N87, SNU-1, SNU-16, AGS, and KATO-III cells were obtained from American Type Culture Collection (Manassas, VA, USA). Briefly, cells were grown in RPMI1640 supplemented with 10% fetal calf serum and 2 mM L-glutamine. Cells were maintained at 37°C in the presence of 5% CO_2_.

Gastric cancer tissues and peripheral blood were collected from patients and healthy controls at the Provincial Hospital (affiliated with Shandong University), after obtaining the subjects' informed consent and with approval from the institutional review board of the hospital. All patients obtained a confirmed diagnosis of gastric carcinoma after resection. Animal experiments were approved by the Institutional Animal Care and Use Committee at Provincial Hospital.

### LncRNA microarray analysis

Total RNA from the samples was quantified using the NanoDrop ND-1000. Microarray analysis was performed using the Agilent Array platform (Agilent Technologies, Santa Clara, CA, USA). Sample preparation and microarray hybridization were performed according to the manufacturer's standard protocols with minor modifications. Briefly, each sample was amplified and transcribed into fluorescent cRNA along the entire length of the transcripts without 3′ bias using a random priming method. The labeled cRNAs were hybridized onto the Human LncRNA Array v2.0 (8 × 60 K, Arraystar). Quantile normalization and subsequent data processing were performed using the GeneSpring GX v11.5.1 software package (Agilent). Differentially expressed lncRNAs and mRNAs with statistical significance were identified through volcano plot filtering and fold-change filtering. Finally, hierarchical clustering was performed based on differentially expressed mRNAs and lncRNAs using Cluster Tree view software (Stanford University, Palo Alto, CA, USA).

### Semi qRT-PCR and qRT-PCR

Total RNA was extracted using Trizol solution. Reverse transcription (RT) was performed in a 20-μL reaction according to the manufacturer's recommendations (Qiagen Inc., Valencia, CA, USA). Semi qRT-PCR and qRT-PCR analyses were performed using the primers listed in Supplementary Table S2. Transcript expression levels were determined by quantifying the intensity of the PCR product normalized to glyceraldehyde-3-phosphate dehydrogenase (*GAPDH*) expression. Quantitative measurement of mRNA levels was performed using the ABI Prism 7000 (Applied Biosystems, Foster City, CA, USA). These data were analyzed using the comparative Ct method.

### Western blotting

Total protein samples were extracted and the concentration was measured using DC protein assay method of Bradford (BD Bioscience, Bedford, MA, USA). A total of 100 μg of protein from each sample was separated by 10% SDS-PAGE and transferred to an equilibrated polyvinylidene difluoride membrane (Amersham Biosciences, Buckinghamshire, UK). Proteins were detected by enhanced chemiluminescence (Amersham Corporation, Arlington Heights, IL, USA) after incubation with primary antibody specific for OR3A4 (1:3,000) or PDLIM2 (1:2,000; Abcam, Cambridge, MA, USA) at 4°C overnight followed by incubation with secondary antibody. Protein levels were normalized to those of total GAPDH using a monoclonal anti-GAPDH antibody (Sigma-Aldrich Corporation, St. Louis, MO, USA) as previously described [4].

### Plasmid vector construction and transfection

The expression vectors for human OR3A4 and PDLIM2 were constructed using PCR methods. The PCR products were confirmed by direct DNA sequencing and cloned into the mammalian expression vectors pEGFP-N1 and pcDNA 3.0 as previously described [3]. The siRNA expression vector, the pSilencer 4.1-CMV/neo plasmid (Invitrogen Corp, Carlsbad, CA, USA), was used for cloning small synthetic oligonucleotides. Two sequences unique to the coding region of OR3A4 were designed and inserted between the BamHI and HindIII sites of the pSilencer 4.1-CMV/neo plasmid. The primers used to construct the three vectors are listed in Supplementary Table S2. MKN-45 and NCI-N87 gastric cancer cell lines were used for overexpression and knockdown studies. For transfection, complexes of Lipofectamine 2000 (Invitrogen) and the desired plasmid were prepared according to the manufacturer's instructions. We obtained stably transfected clones by G418 selection (Promega, Madison, WI, USA). The level of OR3A4 expression after transfection was assayed by RT-PCR. A stable transfectant of the empty vector was used as a control.

### Cell growth and soft agar colony formation

Gastric cancer cells (2 × 10^3^ cells) were incubated with 100 μL of culture medium in 96-multiwell plates for 1 day at 37°C in 5% CO_2_. Cells were transfected with plasmid for 24, 48, 72, 96, and 120 h. Cell number was assessed using the cell-counting kit-8 (CCK-8; Dojindo, Japan). Briefly, CCK-8 (10 μL) was added to each well. After 4 h of incubation at 37°C, absorbance at 450 nm was measured using the ARVO MX plate reader (Perkin Elmer, MA, USA). The number of cells was determined by the relative absorbance at 450 nm.

Gastric cancer cells were trypsinized to yield single-cell suspensions and plated in 6-well plates at a density of 3 × 10^3^ cells/well in complete culture medium containing 0.3% agar layered on top of 0.6% agar. The plates were incubated at 37°C in the presence of 5% CO_2_ for 16 days. Colonies containing at least 50 cells were scored. Data are presented as the mean ± standard deviation of five randomly scored fields.

### Scratch healing, migration, and invasion assays

For the scratch assays, cells were treated with 10 mg/mL mitomycin C (Sigma-Aldrich Corporation, St. Louis, MO, USA) for 3 h and then wounded with a pipette tip. After additing complete medium, the wound closing procedure was observed for 48 h. Photographs were taken every 6 h. For migration and invasion assays, cell culture was performed in Transwell chambers (8 mm, 24-well format; Corning, Painted Post, NY, USA). For the invasion assay, the insert membranes were coated with diluted Matrigel (BD Biosciences, San Jose, CA, USA). Cells (1 × 10^5^) were added to the upper chamber and cultured for 48 h. For the migration assay, the insert membranes were not coated with Matrigel but were cultured under the same conditions. Finally, the insert membranes were removed and stained with crystal violet (Beyotime, Haimen, shanghai, China) and the cells were counted under an inverted microscope and photographed.

### Endothelial tube formation

Endothelial tube formation assays were performed using supernatants from SGC-7901 and NCI-N87 cells. *In vitro* angiogenesis was assessed using an endothelial tube formation assay kit (Cell Biolabs, San Diego, CA, USA). Briefly, each well of a prechilled 96-well culture plate was coated with a thin layer of extracellular matrix (ECM) gel. HUVECs were resuspended in supernatant collected from transfected cells and added to the polymerized ECM gel (2 × 10^4^ cells/300 mL supernatant/well). After an 18 h of incubation, the tube formation ability was evaluated by determining the number of tubules, the tubular length, and the number of tubular intersecting nodes in five random fields using Image Pro Plus software (Media Cybernetics Inc., Bethesda, MD, USA) according to the method of Mirshahi [45].

### Vasculogenic mimicry assay on 3D culture

A 3D culture matrix comprising type I collagen gel and matrigel was produced as follows: 25 mL of type I collagen (BD Bioscience; 3.7 mg/mL) and matrigel (BD Bioscience) were mixed in a 1:1 ratio, dropped onto 16-mm glass cover slides in six-well culture plates, and 100 mL of absolute ethanol was added to each well until the gel polymerized at room temperature. After culture for 4 days without changing the culture media, cells were fixed with 4% formaldehyde in PBS for 10 min. The formation of vasculogenic-like structures was assessed by the presence of Periodic acid-Schiff (PAS)-positive ECM surrounded by cancer cells. Vascular mimicry formation capacity was evaluated by counting the number of vascular mimicking tubules.

### Chick embryo chorioallantoic membrane (CAM) assay

Fertilized chicken eggs (Hua farm, Jinan, China) were incubated at 37°C and 70% humidity. Normally developed embryos were used for the experiments. The open window was sealed with transparent tape and incubated overnight. On the 7th day, a piece of sterilized filter paper disc (0.5 cm diameter) was placed on the surface of the CAM. Each day for 3 consecutive days, 30-μL of supernatant from the experiment and control cell groups was dropped onto the filter paper disc and sealed with transparent tape. On the 10th day of incubation, the eggs were photographed with a MacroPATH dissecting microscope (Milestone Srl, Sorisole BG, Italy) and the number of blood vessels around the filter paper disc was assessed. All vessels vertical to the disc or intersecting with the disc at an angle > 45° were counted.

### Tumor growth in nude mice

Cancer cells transfected with required plasmids were collected (1 × 10^6^ cells in 100 mL) and inoculated subcutaneously into flank regions or injected by direct puncture into the peritoneal cavity of 4-week-old male BALB/c nude mice (Chinese Academy of Sciences, Shanghai, China). Tumor nodules were measured every 4 days with calipers. Tumor growth curves and growth inhibition rates were calculated. The mice were euthanized after 1 month. For the *in vivo* peritoneal dissemination assays, the number of visible nodules in the peritoneal cavity was counted.

### Global cDNA microarray analysis

A whole human genome oligo microarray (Agilent) was used. After hybridization and washing, microarray slides were scanned with an Agilent DNA microarray scanner. The resulting text files extracted from Agilent Feature Extraction Software (version 9.5.3) were imported into the Agilent GeneSpring GX software (version 7.3) for further analysis. Differentially expressed genes were identified through fold-change screening. RT-PCR was performed for target gene verification. The primers for the target genes are listed in Supplementary Table S2.

### RNA immunoprecipitation

RNA immunoprecipitation was performed using a Magna RIPTM RNA-binding protein immunoprecipitation kit (Millipore, Billerica, MA, USA) according to the manufacturer's instructions. Antibodies specific for PDLIM2, MACC1, NTN4, and GNB2L1 (Abcam) were used for RNA immunoprecipitation. Co-precipitated RNAs were detected by RT-PCR. Primers used to detect OR3A4 expression are listed in Supplementary Table S2. Total RNA and controls were also assayed to confirm that the detected signals were from RNAs that specifically bind to PDLIM2, MACC1, NTN4, and GNB2L1.

### RNA pulldown

The RNA pulldown assay was performed using a kit according to the manufacturer's instructions (Roche Diagnostics, Basel, Switzerland). Briefly, biotin-labeled RNAs were transcribed *in vitro* using Biotin RNA Labeling Mix and T7 RNA polymerase, and then treated with RNase-free DNase I. One milligram of SGC-7901 or NCI-N87 cell nuclear extract was mixed with 50 pmol of biotin-labeled RNA, and then 60 μL of washed streptavidin agarose beads (Invitrogen) was added to each binding reaction and further incubated at room temperature for 1 h. Beads were washed briefly five times, boiled in sodium dodecyl sulfate buffer, and the retrieved protein was detected by a standard western blotting technique.

### Flow cytometric analysis of cell cycle

One day before transfection, 1 × 10^6^ gastric cancer cells were seeded into 6-well culture plates without antibiotics. The cells were transfected with a gene expression vector or control vector. Forty-eight hours after transfection, the cells were harvested, fixed in 70% ethanol at −20°C overnight, and then stained with 250 μg/mL propidium iodide (Sigma-Aldrich), 5 μg/mL RNase A (Sigma-Aldrich), and 5 mmol/L EDTA in PBS (pH 7.4) for 30 min. Cell cycle analysis was performed using FACScan (Beckman Instruments, Fullerton, CA, USA).

### Statistical analysis

Differences between groups were analyzed using the Pearson *χ*^2^ test, Student's *t*-test, or ANOVA test. ROC analysis was performed and the area under the curve was calculated to determine the threshold values of low and high OR3A4. Survival curves and time to recurrence were plotted using the Kaplan-Meier method and measured using the log rank test. SPSS 15.0 software (SPSS Inc., Chicago, IL, USA) was used for all statistical analyses and *P* < 0.05 was considered statistically significant. Data are expressed as the mean ± standard deviation from at least three separate experiments.

## SUPPLEMENTARY MATERIALS FIGURES AND TABLES




