# Supplementary material for: Long noncoding RNA OR3A4 promotes metastasis and tumorigenicity in gastric cancer
Source: Oncotarget. 2016 Feb 6;7(21):30276–94. doi: 10.18632/oncotarget.7217 (PMC5058680; doi:10.18632/oncotarget.7217)
Supplement: Supplementary file 1 [file oncotarget-07-30276-s001.pdf]

Long noncoding RNA OR3A4 promotes metastasis and tumorigenicity in gastric cancer

Supplementary Materials

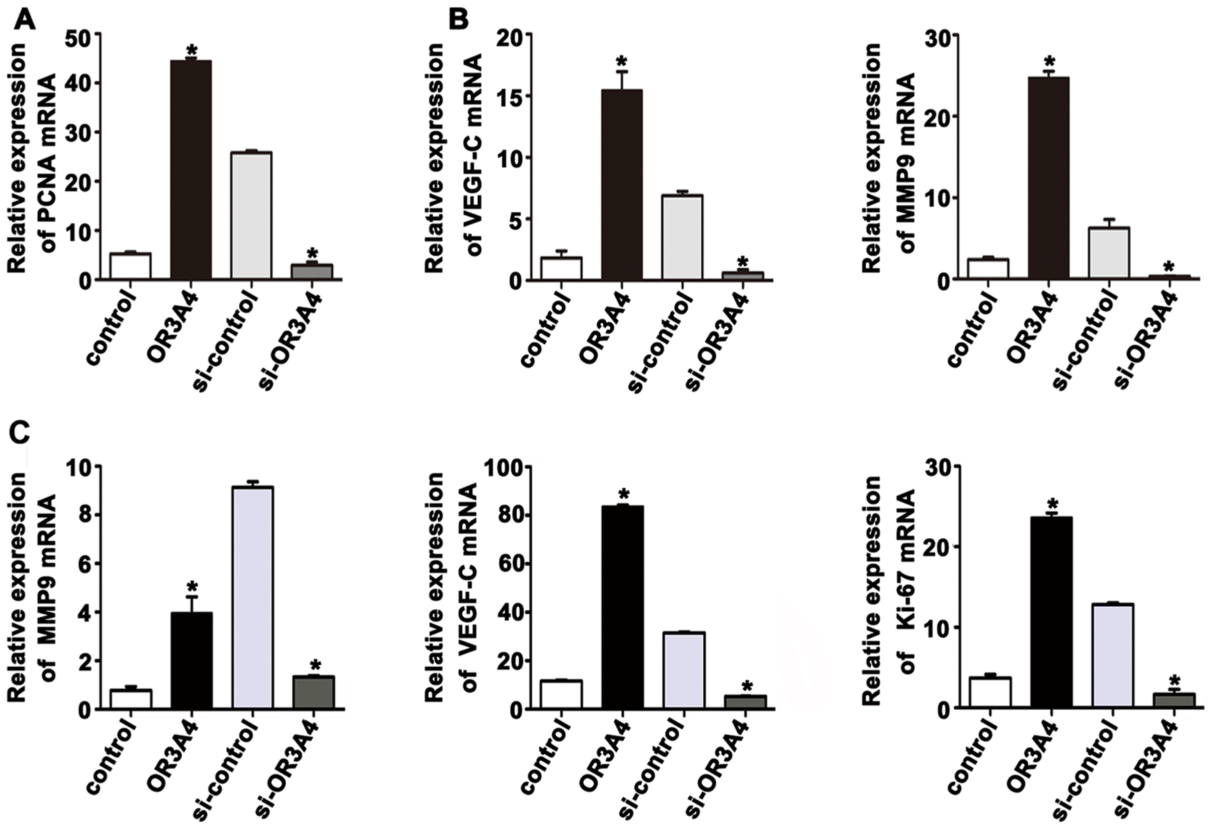

Supplementary Figure S1: Expression levels of genes detected by real-time PCR. (**A**) Expression levels of the *PCNA* gene in NCI-N87 cells. (**B**) Expression levels of the *VEGF-C* and *MMP9* genes in NCI-N87 cells. (**C**) Expression levels of the *MMP9*, *VEGF-C*, and *ki-67* genes in nude mice. *$P < 0.05$. Each bar represents the mean value ± standard deviation from 3 independent experiments.

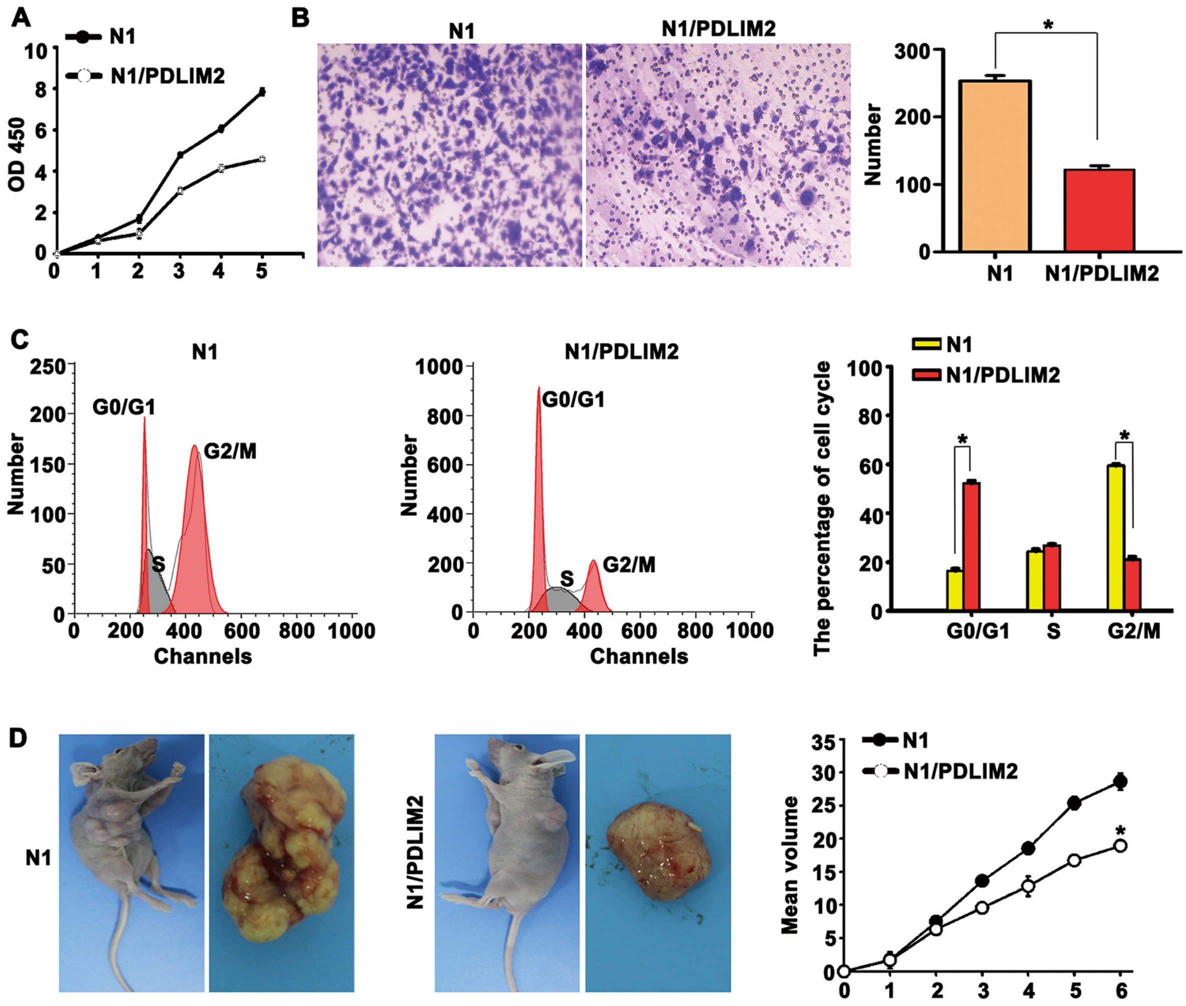

Supplementary Figure S2: Functional validation of PDLIM2 in NCI-N87 cells. We assessed the CCK-8 (**A**) invasion (**B**) cell cycle (**C**), tumor growth in nude mice assay (**D**) upon PDLIM2 overexpression. These results were similar to those described in Figure 7 MKN-45 cancer cells**.**

Supplementary Table S1: Clinicopathological features in gastric cancer patients and normal healthy patients in LncRNA microarray analysis

patients	Age (year)	Gender	Cell differentiation	Tumor size	Gross appearance	Site of tumor	Lymphatic metastasis	Depth of cancer invasion	TNM Stage	Distal metastasis
tumor1	64	Female	Poor differentiation	< 5 cm	Borrmann III	Body	Positive	T4	IV	yes
tumor2	71	Male	Moderate differentiation	< 5 cm	Borrmann III	Body	Positive	T4	IV	yes
tumor3	43	Female	Poor differentiation	≥ 5 cm	Borrmann III	Body	Positive	T4	IV	yes
normal1	42	Male	normal	no	no	no	no	no	no	no
normal2	67	Male	normal	no	no	no	no	no	no	no
normal3	38	Male	normal	no	no	no	no	no	no	no

Supplementary Table S2: Sequences of primers used in this study
